# Different retinopathy phenotypes in type 2 diabetes predict retinopathy progression

**DOI:** 10.1007/s00592-020-01602-9

**Published:** 2020-10-06

**Authors:** Inês P. Marques, Maria H. Madeira, Ana L. Messias, António C.-V. Martinho, Torcato Santos, David C. Sousa, João Figueira, José Cunha-Vaz

**Affiliations:** 1 AIBILI - Association for Innovation and Biomedical Research on Light and Image, Azinhaga de Santa Comba, Celas, 3000-548 Coimbra, Portugal; 2 Faculty of Medicine, Coimbra Institute for Clinical and Biomedical Research (iCBR), University of Coimbra, 3000-548 Coimbra, Portugal; 3 Dentistry Department, Faculty of Medicine, University of Coimbra, 3000-075 Coimbra, Portugal; 4 Ophthalmology Department, Hospital de Santa Maria, 1649-028 Lisbon, Portugal; 5 Vision Sciences Study Center, CECV, Faculdade de Medicina, Universidade de Lisboa, 1649-028 Lisbon, Portugal; 6 Department of Ophthalmology, Centro Hospitalar e Universitário de Coimbra (CHUC), 3000-075 Coimbra, Portugal

**Keywords:** Type 2 diabetes, Retinopathy, Microaneurysm, Retinal thickness, Biomarkers, Phenotypes

## Abstract

**Purpose:**

To characterize the progression in retinopathy severity of different phenotypes of mild nonproliferative diabetic retinopathy (NPDR) in patients with type 2 diabetes.

**Design and methods:**

Patients with type 2 diabetes and mild NPDR (ETDRS 20 or 35) were followed in a 5-year longitudinal study. Examinations, including color fundus photography (CFP) and optical coherence tomography (OCT and OCTA), were performed at baseline, 6 months and then annually. Phenotype classification was performed based on microaneurysm turnover (MAT, on CFP) and central retinal thickness (CRT, on OCT). Phenotype A is characterized by low MAT (< 6) and normal CRT; Phenotype B by low MAT (< 6) and increased CRT; and Phenotype C by higher MAT (≥ 6) with or without increased CRT. ETDRS grading of seven fields CFP was performed at the initial and last visits.

**Results:**

Analysis of ETDRS grade step changes showed significant differences in diabetic retinopathy (DR) progression between the different phenotypes (*p* < 0.001). Of the 66 participants with phenotype A only 2 eyes (3%) presented 2-or-more-step worsening. None of the 50 participants characterized as phenotype B developed 2-step worsening, whereas 13 eyes (23.2%) characterized as phenotype C had 2-or-more-steps worsening. Phenotype C presents the higher risk for 2-or-more step worsening (OR: 15.94 95% CI: 3.45–73.71; *p* < 0.001) and higher sensitivity, correctly identifying 86.7% of cases at risk (AUC: 0.84 95% CI: 0.72–0.96; *p* < 0.001). Diabetic retinopathy severity progression was associated with HbA_1c_ (*p* = 0.019), LDL levels (*p* = 0.043), and ocular factors as MAT (*p* = 0.010), MA formation rate (*p* = 0.014) and MA disappearance rate (*p* = 0.005). Capillary closure at 5-year follow-up, identified by lower vessel density (VD) on OCTA, was also associated with diabetic DR severity progression (*p* = 0.035).

**Conclusions:**

Different DR phenotypes in type 2 diabetes show different risks of retinopathy progression. Phenotype C is associated with increased HbA_1c_ values and presents a higher risk of a 2-or-more-step worsening of the ETDRS severity score.

## Introduction

Diabetic retinopathy (DR) is one of the leading causes of blindness worldwide [1] and the prevalence of vision-threatening DR is expected to double in the next decade [2]. Considering that more than 90% of cases of vision loss can be prevented [3], accurate staging and classification of DR are paramount to guide treatment decision and determine prognosis. The early treatment of diabetic retinopathy study (ETDRS) severity score is the gold standard for DR staging [4]. However, it is labor-intensive and has limited applicability outside of the research setting.

Our group has reported on 2-year and 3-year follow-up studies of people with type 2 diabetes and mild nonproliferative diabetic retinopathy (NPDR) and found marked individual variations in the progression of DR and development of vision-threatening complications. Using non-invasive imaging methodologies (color fundus photography and optical coherence tomography), we have identified three different phenotypes of NPDR. These are based on microaneurysm turnover (MAT) and central retinal thickness (CRT), being associated with that show different risks for development of vision-threatening complications [5].

**Fig. 1:**
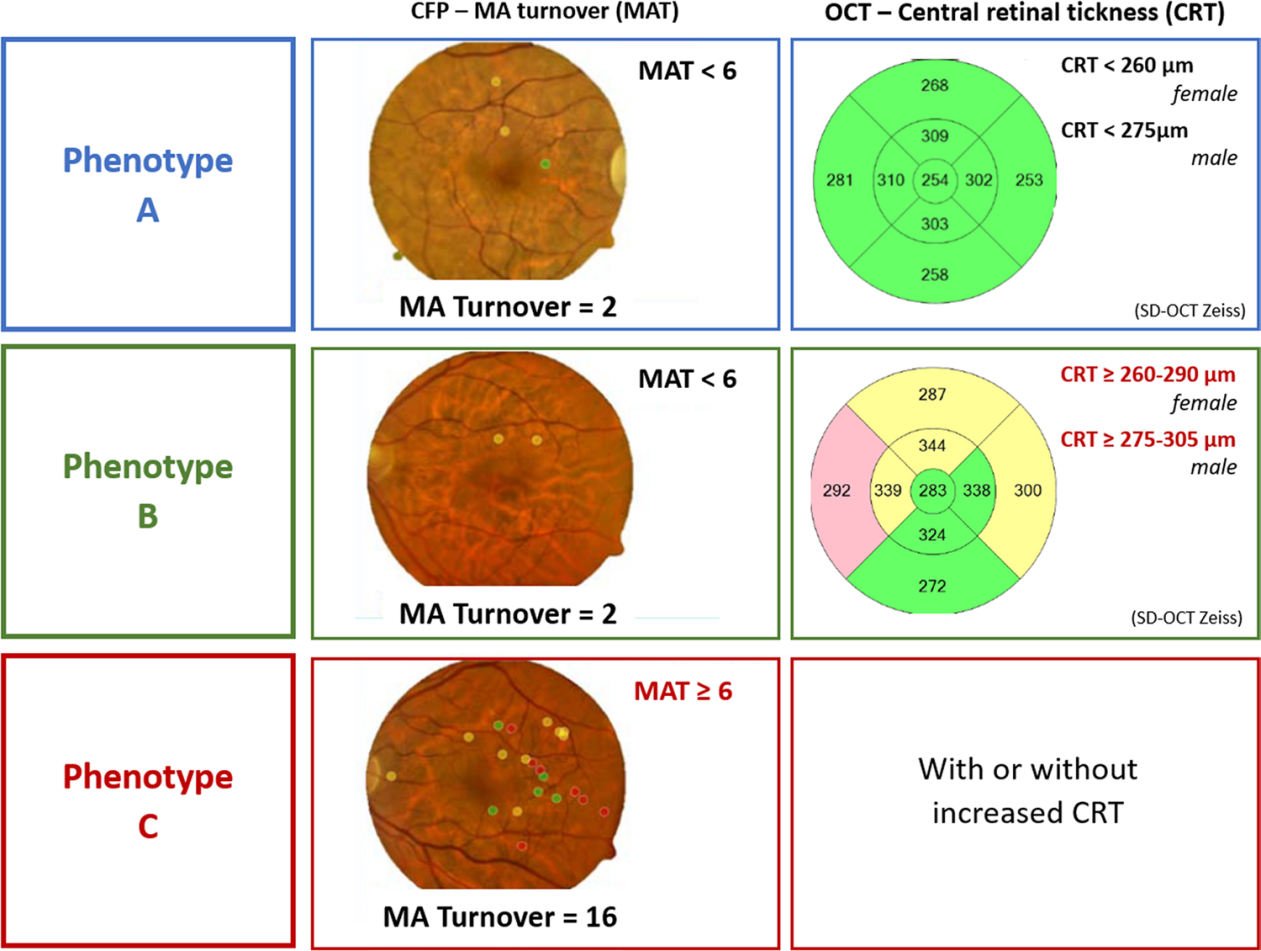
Representative cases for the three phenotypes of DR progression (color fundus image: MA earmarked using the software RetmarkerDR at 6 months visit: red dots are new MA, yellow dots are MA that disappeared from baseline to V6months, and green dots are MA that were present in both visits; Central Retinal Thickness: central macular thickness maps obtained with the SD-OCT). Reference values from Zeiss SD-OCT

Briefly, phenotype A is characterized by low MAT (< 6) and normal CRT; Phenotype B by low MAT (< 6) and increased CRT; Phenotype C by higher MAT (≥ 6) with or without increased CRT (Fig. 1).

A recently published study revealed that these different retinopathy phenotypes in type 2 diabetes show different 5-year risk for development of center-involved macular edema (CIME), clinically significant macular edema (CSME) and/or proliferative diabetic retinopathy (PDR). Phenotype C eyes are at higher risk for development of vision-threatening complications (CSME or PDR). It is also the only phenotype associated with PDR. In contrast, phenotype A identifies eyes that are at very low risk of development of vision-threatening complications [6].

This 5-year longitudinal study of a large cohort of people with type 2 diabetes and mild NPDR aims to identify risk markers for DR severity progression using only non-invasive examination methodologies—digital color fundus photography (CFP), optical coherence tomography (OCT) and OCT-angiography (OCTA).

## Methods

A prospective longitudinal cohort study was designed to follow eyes with minimal or mild NPDR (ETDRS grades 20–35) for a 5-year period or until the time of development of diabetic macular edema (DME) or PDR [6]. The tenets of the Declaration of Helsinki were followed, and the approval was obtained from the local Institutional Ethical Review Board with the number CEC/007/16. Each participant signed a written informed consent, agreeing to participate in the study, after all procedures were explained.

A total of 212 patients with diagnosed adult-onset type 2 diabetes and NPDR grade ETDRS 20–35 were included, with a maximum glycated hemoglobin A_1c_ (HbA_1c_) value of 10%.

Exclusion criteria included any previous laser treatment or intravitreal injections, presence of age-related macular degeneration, glaucoma, vitreomacular disease and high ametropia (spherical equivalent greater than − 6 and +2 DPT), or any other systemic disease that could affect the eye, with special attention for uncontrolled systemic hypertension and history of ischemic heart disease. Eyes with baseline central retinal thickening identifying CIME (defined as a CRT ≥ 290 μm in women and ≥ 305 μm in men, by the Diabetic Retinopathy Clinical Research Network [7]), were excluded.

At baseline visit (V0) demographics, such as age, duration of diabetes and concomitant medications were collected for each participant. Physical assessment with biometric measures (body weight and height) and blood pressure evaluation was performed by an experienced nurse, as well as blood analysis with determination of HbA_1c_ and lipid profile. The remaining study visits were performed at 6 months (V1), 12 months (V2), 24 months (V3), 36 months (V4), 48 months (V5) and 60 months (V6) or last visit before treatment. At all study visits, patients underwent a complete eye examination, which included best correct visual acuity (BCVA, measured for each eye using the ETDRS protocol and Precision Vision charts at 4 m), slit-lamp examination, intraocular pressure (IOP) measurement, digital seven field CFP and OCT. OCTA was performed only at last visit.

The study eye was selected at baseline based on the inclusion/exclusion criteria. When both eyes fulfilled the criteria, the eye showing the more advanced ETDRS grading in any given patient was chosen to be the study eye.

The patients did not receive any treatment during the follow-up period, as defined in the protocol of the study. Treatments were only applied when an outcome was developed, and therefore, the patient left the study.

## Color fundus photography and RetmarkerDR: microaneurysm quantification

CFP was performed according to the ETDRS protocol. The seven fields photographs were obtained at 30/35°, using a Topcon TRC 50DX camera (Topcon Medical Systems, Tokyo, Japan). The DR severity level was determined by two independent graders within the context of an experienced reading center (Coimbra Ophthalmology Reading Center - CORC, Coimbra, Portugal) and was based on the seven field protocol according to the modified Airlie House classification of diabetic retinopathy used by the ETDRS [4]. According to the ETDRS final scale (ETDRS report number 12), a severity scale was used by identifying step changes. The DR severity scale used was given as follows: step 1, level 10, no retinopathy; step 2, level 20; step 3, level 35; step 4, level 43; step 5, level 47; step 6, level 53; step 7, levels 61 to 71.

Additionally, 45/50° field-2 images were obtained and subjected to automated microaneurysm (MA) analyses using the RetmarkerDR (Retmarker SA, Coimbra, Portugal). This automated computer-aided diagnostic system consists of software earmarking MA and red-dot-like vascular lesions in the macula (all referred to as MAs); it includes a co-registration algorithm that allows comparison within the same retina location between different visits for the same eye, as previously described [8, 9]. Briefly, the algorithm computes for each eye the number of MAs in each visit, the number of MAs that appear and/or disappear from one visit to the other, allowing calculation of the number of MAs appearing and/or disappearing per time interval (i.e., the MA formation rate and the MA disappearance rate, respectively). The MAT is computed as the sum of the MA formation and disappearance rates, determined at 6-month visit.

## Characterization of retinopathy phenotypes

The three different DR phenotypes for NPDR, previously described by our group [5, 10], were identified in the study eyes at the 6-month visits, based on the six-month MAT and CRT, and according to the following rules: Phenotype A: MAT < 6 and normal CRT values (central subfield RT < 260 µm for females and < 275 µm in males, i.e., normal mean ± 1 SD); Phenotype B: MAT < 6 and increased CRT values (CRT ≥ 260 µm for females and ≥ 275 µm in males); Phenotype C: MAT ≥ 6, with or without increased CRT. Central retinal thickness reference values presented are the reference for Zeiss Cirrus SD-OCT [11, 12].

## Optical coherence tomography

OCT was performed using the Cirrus Zeiss 5000 AngioPlex (Carl Zeiss Meditec, Dublin, CA).

The Macular Cube 512 × 128 acquisition protocol, consisting of 128 B-scans with 512 A-scans each, was used to assess the subjects’ CRT, collected from the standard Cirrus examination reports. Segmentation of retinal layers to assess the average thickness value of the ganglion cell layer plus inner plexiform layer (GCL + IPL) in both central subfield (CSF) and Inner Ring (InRing) were performed using the segmentation software implemented by AIBILI [6, 13]. Automated analysis results were reviewed by a masked grader.

Eyes with CIME were identified following the reference values established by the DRCR.net for Cirrus SD-OCT [7]. GCL + IPL thickness decreases were considered as a surrogate marker of neurodegeneration [14] whereas full CRT increases were considered to identify edema [15], comparing to a healthy control population [13, 16].

## OCT-angiography

OCTA was performed in all patients but only in the last study visit. OCTA data were collected by the CIRRUS™ HD-OCT 5000 with AngioPlex^®^ OCT-angiography (Carl Zeiss Meditec, Dublin, CA, USA) device using Angiography 3x3 mm acquisition protocol, which consists of a set of 245 clusters of B-scans repeated four times, where each B-scan consists of 245 A-scans over a 3x3x2 mm cube in the central macula.

To calculate vessel density (VD) a thresholding algorithm was applied to the superficial and deep capillary layers *en*-*face* images to create a binary slab that assigns to each pixel a 1 (perfused) or 0 (background). From this slab, a skeletonized slab was created, representing vessels with a trace of 1 pixel in width as described in [17]. We define VD as skeletal density being the total length of perfused vasculature per unit area in a region of measurement. A similar length-based metric has been used as a measurement of road density. We calculate VD by taking the mean of the skeletonized slab within a region of interest and scaling the result by the distance between pixels (in this case, 245 pixels per 3 mm).

For quality check, all OCTA acquisitions were reviewed by a masked grader. Only eyes that had OCTA examinations with signal strength greater or equal to 7, minimal motion artifacts and no evidence of defocus or blur were included in the analysis.

## Outcomes and statistical analysis

The collected data on each eye/patient is presented as means and corresponding standard deviations for continuous variables or absolute and relative frequencies for categorical and ordinal variables. ETDRS step changes were determined as the difference between levels of ETDRS at baseline and at the 5 years follow-up and classified as improvement or worsening according to the reduction or increase of the retinopathy grade. Classification included “2-step improvement,” “1-step improvement,” “maintenance,” “1-step worsening” and “2-or-more-step worsening.” Due to the clinically less relevant 1-step change, patients with 1-step improvement and 1-step worsening were gathered under the category of maintenance—Maintenance (± 1 step deviation).

Comparison of baseline characteristics of patients with different ETDRS changes over the 5 years of follow-up was performed using the Kruskal–Wallis test (due to violation of assumption of normality) and all-pairwise post hoc comparisons with Bonferroni correction or the Chi-square test with Monte-Carlo correction.

A multinomial logistic regression was run to assess the likelihood of 2-step improvement or 2-or-more steps worsening associated with each demographic, systemic and ocular marker evaluated at the baseline or 6 months appointment—MAT, MA formation rate, MA disappearance rate, CRT, GCL + IPL CSF thickness, GCL + IPL InRing thickness, capillary closure metrics and phenotype. Results were presented as odds ratios (OR) and corresponding 95% confidence intervals. The significant predictors (*p* < 0.05) were considered for multivariate analysis, and the obtained predicted probabilities were tested for the discriminatory performance using receiver operating characteristic (ROC) curves.

All analyses were performed with SPSS (IBM SPSS Statistics version 24; IBM Corp ©, New York, USA), and a *p* value < 0.05 was considered statistically significant.

## Results

Of the 212 eyes/patients included in the study, 172 completed the 5-year follow-up or achieved one of the endpoints, CSME, CIME or PDR. Forty patients dropped out of the study (nine died, twenty-one withdrew from the study and ten were lost to follow-up). The eyes included in the study had mild NPDR, 58 (27%) graded as ETDRS level 20 and 154 (73%) graded as ETDRS level 35. As previously described, no statistically significant differences were found for demographic, clinical and ocular characteristics at baseline between the 172 patients that reached the study endpoint or performed the last visit of the study and the eyes/patients that dropped out from the study [6].

The distribution of the ETDRS change over the 5-year period for the 172 eyes that completed the study is detailed in Table 1. Twelve eyes (7%) presented 2-step improvement and 15 (9%) had 2-or-more-step worsening of their retinopathy severity. The vast majority maintained the ETDRS classification (*n* = 77) or presented 1-step variation: 1-step improvement (*n* = 19) and 1-step worsening (*n* = 49).

**Table 1:** Baseline characteristics of the population with different ETDRS changes over the 5-year period

Characteristics	All patients included	2-step improvement	Maintenance	2-or-more-step worsening	*p*
Demographics					
Males/females, *n* (%)	117 (68.0)/55 (39.0)	9/3 (75/25)	98/47 (67.6/32.4)	10/5 (66.7/33.3)	0.891
Age, mean ± SD, y	62.7 ± 7.2	61.3 ± 7.1 (60.5)	62.9 ± 7.1 (63.0)	62.1 ± 8.4 (59.0)	0.683
Diabetes duration, mean ± SD, y	14.2 ± 7.4	14.1 ± 9.5 (12.50)	14.2 ± 7.4 (14.0)	14.3 ± 6.2 (15.0)	0.993
Clinical characteristics, mean ± SD (median)					
BMI, kg/m^2^	30.1 ± 5.9 (29.7)	31.9 ± 8.1 (30.6)	29.8 ± 5.6 (29.3)	31.4 ± 5.9 (31.2)	0.478
HbA_1c_, %	7.5 ± 1.3 (7.4)	6.9 ± 0.7 (6.5)	7.5 ± 1.3 (7.4)	8.3 ± 1.4 (8.0)	0.025*
Total cholesterol, mg/dL	184.0 ± 38.6 (181.5)	194.4 ± 20.9 (193.0)	185.0 ± 38.8 (181.0)	167.1 ± 46.2 (160.0)	0.198
HDL cholesterol, mg/dL	47.4 ± 11.1 (47.0)	47.9 ± 12.5 (45.0)	47.4 ± 10.8 (46.5)	47.9 ± 12.9 (48.0)	0.893
LDL cholesterol, mg/dL	122.2 ± 32.8 (118.5)	127.6 ± 19.8 (130.0)	123.6 ± 32.9 (119.0)	105.4 ± 35.8 (109.0)	0.130
Triglycerides, mg/dL	166.4 ± 93.5 (142.0)	175.3 ± 78.5 (176.0)	164.6 ± 89.7 (144.5)	176.2 ± 135.9 (125.0)	0.692
Systolic BP, mmHg	138.1 ± 15.9 (139.0)	129.8 ± 15.7 (131.5)	138.4 ± 16.1 (140.0)	141.9 ± 11.7 (143.0)	0.206
Diastolic BP, mmHg	71.9 ± 9.0 (72.0)	70.1 ± 8.5 (68.0)	72.0 ± 9.1 (72.0)	73.1 ± 8.3 (72.0)	0.697
Ocular characteristics, mean ± SD (median)					
BCVA, letters	85.6 ± 3.9 (85.09	86.6 ± 3.2 (87.0)	85.5 ± 3.9 (85.0)	85.4 ± 4.7 (85.0)	0.484
MA turnover, no. per 6 months	7.0 ± 12.5 (3.7)	1.6 ± 2.5 (0.0)	6.2 ± 11.5 (3.6)	19.7 ± 18.3 (15.6)	<0.001*
MA formation rate, no. per 6 months	3.1 ± 6.4 (1.9)	0.3 ± 1.1 (0.0)	2.7 ± 5.8 (1.9)	8.9 ± 10.9 (7.8)	< 0.001*
MA disappearance rate, no. per 6 months	3.9 ± 6.7 (2.0)	1.3 ± 1.7 (0.0)	3.5 ± 6.3 (2.0)	10.8 ± 8.8 (8.3)	< 0.001*
CRT, µm	266.8 ± 21.7 (267.0)	268.6 ± 18.4 (267.5)	267.3 ± 22.0 (268.0)	260.4 ± 20.6 (256.0)	0.314
GCL + IPL CSF thickness, µm	39.1 ± 9.5 (38.6)	40.4 ± 6.2 (40.2)	39.4 ± 9.9 (39.1)	35.5 ± 6.6 (33.9)	0.118
GCL + IPL InRing thickness, µm	90.8 ± 10.0 (91.2)	95.2 ± 6.4 (93.1)	90.7 ± 9.9 (91.2)	88.1 ± 12.0 (84.2)	0.106
Baseline ETDRS level, n(%)					
20	48 (27.9)	0 (0.0)	45 (31.0)	3 (20.0)	0.056
35	124 (72.1)	12 (100.0)	100 (69.0)	12 (80.0)	
Phenotypes, n(%)					
Phenotype A	66 (38.4)	6 (9.1)	58 (87.9)	2 (3.0)	< 0.001*
Phenotype B	50 (29.1)	5 (10.0)	45 (90.0)	0 (0.0)	
Phenotype C	56 (32.6)	1 (1.8)	42 (75.0)	13 (23.2)	

*BMI* body mass index, *HbA*_*1c*_ hemoglobin A_1C_, *HDL* high-density lipoprotein, *LDL* low-density lipoprotein, *BP* blood pressure, *BCVA* best corrected visual acuity, *MA* microaneurysm, *CRT* retinal thickness, *GCL* ganglion cell layer, *IPL* inner plexiform layer, *CSF* central subfield, *InRing* inner ring, *ETDRS* early treatment diabetic retinopathy study

*And bold values represent statistically significant alterations, with *p* < 0.05

Among the demographic and clinical characteristics, statistically significant differences across the categories of ETDRS change were only found for HbA_1c_ (*p* = 0.025), for which was possible to register a continuous increase in the mean and median values from 2-step improvement to 2-or-more-step worsening. Pairwise comparisons indicated that the difference was set between those with 2-step improvement and 2-or-more-step worsening (*p* = 0.024) (Table 1).

MAT, MA formation rate and MA disappearance rate presented statistically significant differences across ETDRS change categories (*p* < 0.001 for the three variables), with an increase in the median values from 2-step improvement to 2-or-more-step worsening. Pairwise comparisons indicated that the eyes with 2-or-more-step worsening presented statistically higher MAT than any other category (2-or-more-step improvement: *p* < 0.001; maintenance: *p* = 0.036).

**Fig. 2:**
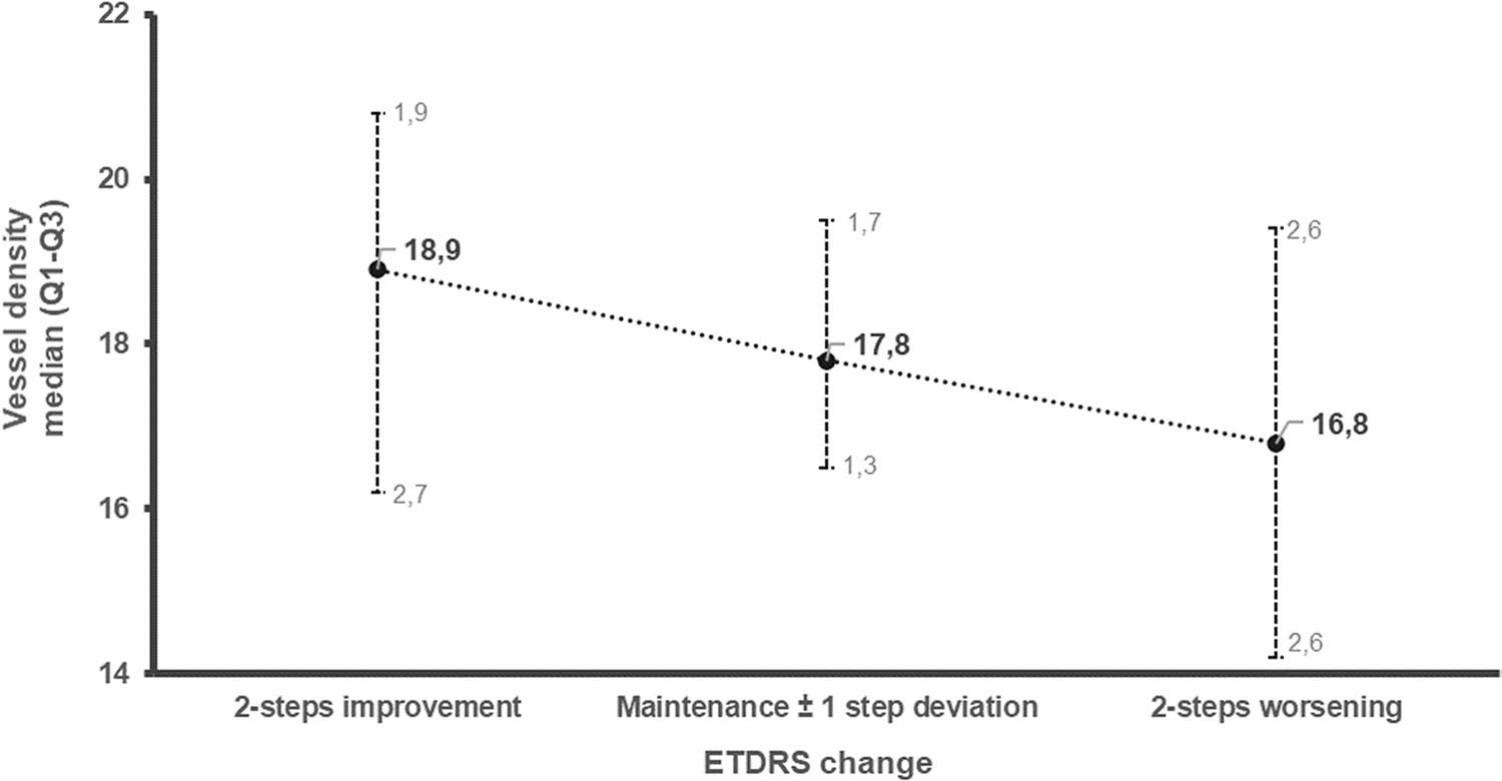
Correlation between vessel density at last visit with ETDRS step change over time. Vessel density was assessed at last visit of the study and is presented as Median and quartile 1 and 3 range. ETDRS step change was accessed by the difference between ETDRS grades in in the baseline and last visit, considering step improvement, maintenance or worsening and represents diabetic retinopathy progression

Vessel density obtained from the OCT-A at the last visit of the 5-year follow-up showed a decrease in all ETDRS levels. This decrease indicates a significant correlation with the DR severity progression. A consistent decrease in the median values of VD, obtained from the OCTA at 5-year of follow-up, was found when comparing the two-step worsening category (median: 16.8; interquartile range [IQR]: 2.6–2.7) with the improvement category (median: 18.9; IQR: 1.9–2.7) (Fig. 2).

At the 6-month visit, the eyes were categorized as phenotype *A*, *B* or *C*, as detailed in the methods section. The distribution of ETDRS changes across phenotypes is detailed in Table 1. During the 5-year period of follow-up, only three eyes (3%) of phenotype A presented 2-or-more-step worsening. In phenotype B, there were no cases of 2-or-more-step worsening. Finally, in phenotype C a total of 13 eyes (23%) presented 2-or-more-step worsening in the ETDRS score. These results indicate a non-independent distribution of ETDRS changes across the three phenotypes (*p* < 0.001) with higher prevalence of 2-or-more-step worsening associated with phenotype C. The univariate multinomial regression corroborated those patients that have 2-or-more-step worsening present particular features which are not observed in the patients falling into the other categories. While, it was possible to identify higher HbA_1c_ levels (OR = 1.66, 95% CI: 1.09–2.54), lower LDL cholesterol (OR = 0.98, 95% CI: 0.96–1.00), and elevated MAT (OR = 1.05, 95% CI: 1.01–1.09), MA formation rate (OR = 1.08, 95% CI: 1.02–1.15) and MA disappearance rate (OR = 1.10, 95% CI: 1.03–1.18) as significant univariate risk predictors of 2-or-more-step worsening when compared to maintenance (± 1 step deviation), no factor was identified as predictor of 2-step improvement (Table 2). Phenotype C was associated with a significantly 16-fold higher risk of 2-or-more-step worsening (OR = 15.94, 95% CI: 3.45–73.71) while no phenotype was associated with an increased risk of improvement ETDRS score during follow-up.

**Table 2:** Univariate multinomial regression of demographic, systemic and ocular predictors of ETDRS progression over the 5-year period, referencing to maintenance (± 1-step deviation)

	2-step improvement		2-or-more-step worsening	
	OR (95% CI)	*p*	OR (95% CI)	*p*
Demographic features				
Age	0.97 (0.89–1.05)	0.440	0.98 (0.92–1.06)	0.577
Diabetes duration	0.97 (0.92–1.08)	0.968	1.00 (0.93–1.08)	0.936
Gender (male)	1.44 (0.37–5.56)	0.598	0.96 (0.31–2.96)	0.942
BMI	1.06 (0.96–1.17)	0.239	1.05 (0.96–1.14)	0.325
Systemic features				
HbA_1c_	0.65 (0.38–1.12)	0.117	1.66 (1.09–2.54)	0.019*
Total cholesterol	1.01 (0.99–1.02)	0.442	0.99 (0.97–1.00)	0.088
HDL	1.00 (0.95–1.06)	0.874	1.01 (0.96–1.05)	0.849
LDL	1.00 (0.99–1.02)	0.692	0.98 (0.96–1.00)	0.042*
Triglycerides	1.00 (1.00–1.01)	0.712	1.00 (1.00–1.01)	0.646
Systolic blood pressure	0.96 (0.93–1.00)	0.076	1.01 (0.98–1.05)	0.412
Diastolic blood pressure	0.98 (0.91–1.05)	0.481	1.01 (0.96–1.07)	0.634
Phenotypes				
Phenotype A	1.50 (0.46–4.88)	0.500	0.23 (0.05–1.06)	0.060
Phenotype B	1.59 (0.48–5.27)	0.451	Not possible to compute^a^	
Phenotype C	0.22 (0.03–1.78)	0.157	15.94 (3.45–73.71)	<0.001*
Ocular markers				
MA turnover	0.76 (0.58–1.00)	0.049	1.05 (1.01–1.09)	0.010*
MA formation rate	0.52 (0.26–1.02)	0.058	1.08 (1.02–1.15)	0.014*
MA disappearance rate	0.78 (0.57–1.06)	0.116	1.10 (1.03–1.18)	0.005*
CRT	1.00 (0.98–1.03)	0.837	0.99 (0.96–1.01)	0.245
GCL + IPL CSF thickness	1.01 (0.95–1.08)	0.720	0.96 (0.90–1.01)	0.126
GCL + IPL InRing thickness	1.06 (0.99–1.14)	0.103	0.98 (0.93–1.02)	0.345
Ratio GCL + IPL/RT (CSF)	0.84 (0.56–1.27)	0.409	1.09 (0.84–1.42)	0.530

*BMI* body mass index, *HbA*_*1c*_ hemoglobin A_1C_, *HDL* high-density lipoprotein, *LDL* low-density lipoprotein, *MA* microaneurysm, *CRT* retinal thickness, *GCL* Ganglion cell layer, *IPL* inner plexiform layer, *CSF* central subfield, *InRing* inner ring, *RT* retinal thickness

^a^Zero cases of phenotype B presented two steps worsening in the ETDRS classification over the 5 years

*And bold values represent statistically significant alterations, with *p* < 0.05

**Fig. 3:**
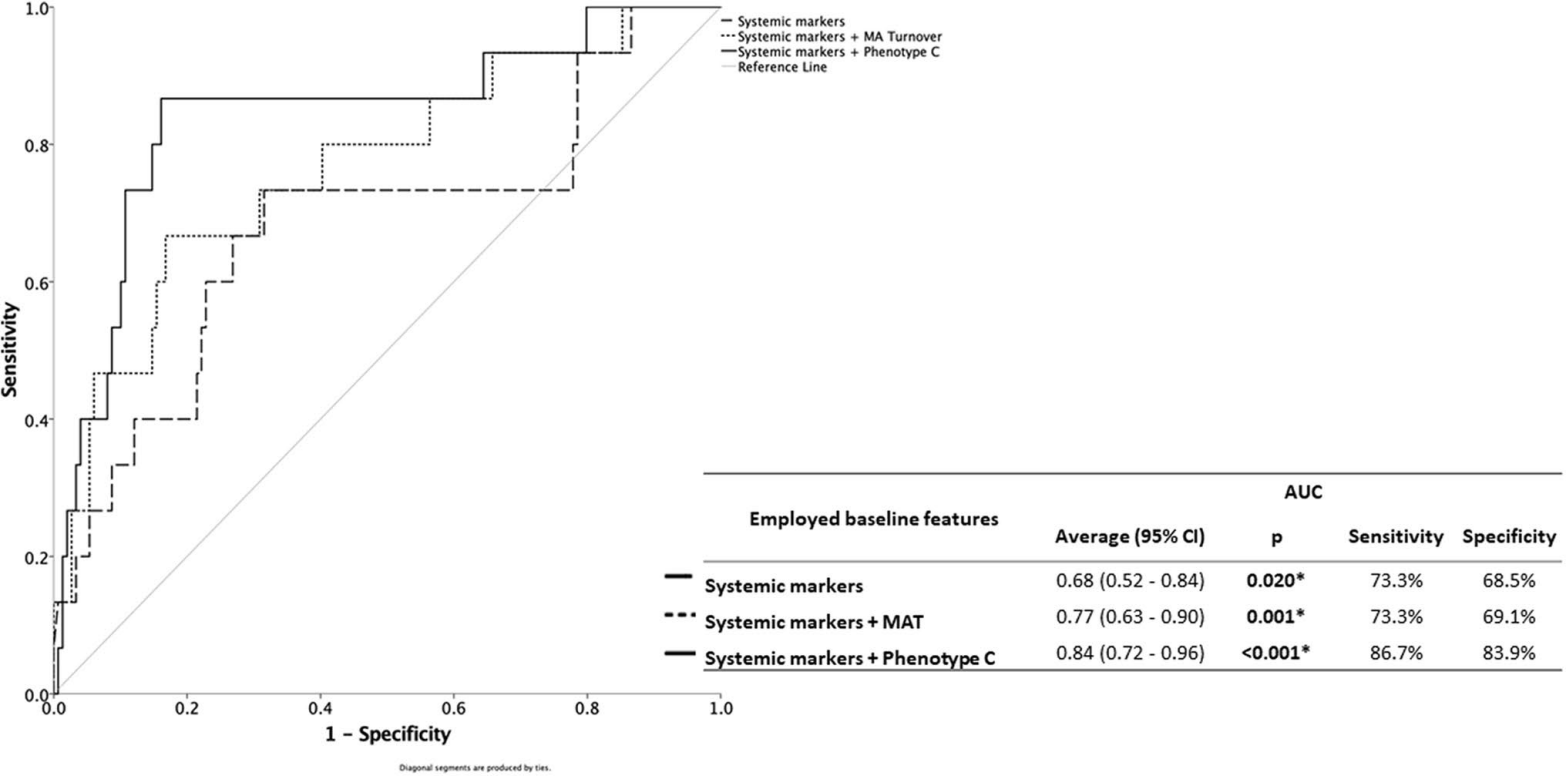
ROC curve for Systemic markers, Systemic markers + MAT and Systemic markers + Phenotype C on the prediction of 2-or-more ETDRS grade step worsening. Includes indication of AUC (95% confidence interval), sensitivity and specificity

Two multivariate analyses were performed (Table 3) using important metabolic systemic factors, HbA_1c_ and LDL cholesterol. In one case, the analysis included MAT (adjusted OR = 1.04, 95% CI: 1.00–1.08), whereas in the other case the analysis included phenotype C (adjusted OR = 12.23, 95% CI: 2.53–59.18). Though both models presented good discriminatory capacity of determining 2-or-more-step progression with area under the curve (AUC) > 75%, the model considering MAT could not increase the sensitivity of the systemic markers alone model. Contrarily, phenotype C, presented higher sensitivity, correctly identifying 87% of cases at risk of 2-or-more-step progression, and good specificity, correctly determining 84% of the cases not at risk (Fig. 3).

**Table 3:** Multivariate regression analysis of 2-or-more-step worsening risk

	OR (95% CI)	*p*
Systemic features		
HbA_1c_	1.83 (1.09–3.07)	0.021*
LDL	0.97 (0.95–0.99)	0.010*
Ocular markers		
MA Turnover	1.04 (1.00–1.08)	0.033*
Systemic features		
HbA_1c_	1.66 (0.98–2.81)	0.057
LDL	0.98 (0.96–1.00)	0.021*
Phenotypes		
C	12.23 (2.53–59.18)	0.002*

*HbA*_*1c*_ hemoglobin A_1C_, *LDL* low-density lipoprotein, *MA* microaneurysm

*And Bold values represent statistically significant alterations, with *p* < 0.05

## Discussion

This 5-year prospective longitudinal study of patients with type 2 diabetes and mild NPDR (ETDRS 20 and 35 at baseline) shows that phenotype C, is a good predictor of retinopathy worsening as demonstrated by 2-or-more-step increase in ETDRS score. Of particular interest is the observation that MAT determined over a period of only 6 months, predict, with a high degree of confidence, the eyes that do not progress for a period of at least 5 years. This finding potentially may have impact in clinical trial design allowing programmed recruitment and in the clinical management of the initial stages of diabetic retinal disease.

We know from previous epidemiological studies that the incidence of clinically significant endpoints (e.g., PDR or CSME), is very low in patients with mild NPDR. For that reason, clinical trials based on these primary endpoints require a long period of follow-up and a large number of patients to include. Thus, other clinically meaningful measures have been proposed as primary endpoints, such as 2 or 3-step progression on the ETDRS DR severity scale [18] or change in MA counts [19]. Although of some complexity, the ETDRS DR severity scale has become the “de facto” gold standard for grading diabetic retinopathy and any evaluation of progression in severity of diabetic retinopathy should refer to it. Indeed, recent studies have shown the relevance of retinopathy severity evaluation based on ETDRS grading as a clinically important outcome. In eyes treated with anti-VEGF agents [20] or with corticosteroids [21], greater degrees of improvement in ETDRS score correlate with greater magnitudes of functional and anatomic improvement. In fact, 2-step worsening in ETDRS grades has been accepted as clinically relevant and allowing to use smaller number of patients or shorten the duration of the trial [22]. However, ETDRS classification has some limitations to be used in routine screening of DR patients in clinic, as it is difficult to perform and time consuming.

The present study shows that automated analysis of MAT correlates well with changes in ETDRS severity levels validating its use as a simple to use biomarker of DR progression. Automated analysis techniques offer advantages of repeatability and consistency associated with ease of use. It is also relevant that MAT calculated by the RetmarkerDR is much less time consuming than ETDRS grading and only needs a CFP to be performed.

Increased values of MAT and phenotype C, independent of CRT values, appear to identify the eyes that will be progressing and developing vision-threatening complications such as CSME and PDR, which are not expected to improve without intervention. Our observations suggest that the eyes/patients that can be identified by these methods are the ones that need most close follow-up.

Phenotype C was identified mainly in eyes with baseline ETDRS level 35 (97%) suggesting that ETDRS level 35 may be the turning point in the progression of diabetic retinopathy. Eyes with ETDRS level 35 apparently reach a status of microvascular damage that creates the conditions for either stabilization or progression demonstrated by identification of Phenotype C. In this study approximately 44% of the eyes graded as ETDRS level 35 at baseline were classified as phenotype C. Of the eyes classified as phenotype C 23% experienced a 2-or-more steps ETDRS grade worsening of their retinopathy severity during the 5-year period of follow-up; whereas for patients classified as phenotype A or B only 2% presented a 2-or-more steps grade worsening.

We have also observed a correlation between capillary closure, identified by decreased VD and retinopathy severity progression, indicating that this OCTA metric is a potential early marker of DR severity progression. An automated non-invasive examination such as OCTA offers a promising option to identify retinopathy progression [16].

When considering systemic variables HbA_1c_ stands out as being associated with retinopathy severity progression. It is the systemic variable that shows a clearer association with retinopathy progression.

Our study identified a large group of eyes/patients, phenotypes A and B, which combined represent 70% of the entire cohort and which are at a very low risk for 2-or-more-step ETDRS worsening (2%). This observation is particularly relevant for appropriate planning of eye care for the large numbers of patients with type 2 diabetes and mild retinopathy.

Limitations of this study include the focus on the initial stages of DR, allowing conclusions to be made only on the progression of ETDRS grades 20–35. The fact that there was no correction of the VD according to signal strength differences between 7 and 10 is another limitation of the study, as it has been suggested that differences in VD can be found according signal strength, and quantification algorithms for OCTA should ideally remove the signal strength bias [23].

However, the 5-year prospective follow-up is a major strength as it offers new insights into the progression of retinal diabetic disease, particularly when it may still be reversible and amenable to treatment. Fundus photography, including MAT evaluation using the RetmarkerDR, OCT and OCTA are easy to perform and can be repeated without major inconvenience to the patient or clinics’ flow. This study confirms the potential of these variables for the evaluation of DR severity progression, opening new avenues for improved management strategies of NPDR and timely identification of eyes at risk for retinopathy progression.
